# Spontaneous Air Embolism Following Contrast Injection: A Diagnostic Challenge in a Post-COVID-19 Patient

**DOI:** 10.7759/cureus.67375

**Published:** 2024-08-21

**Authors:** Raed Al Yacoub, Michael Ladna, Zaid Al-Radideh, Johnny F Jaber

**Affiliations:** 1 Internal Medicine, University of Florida College of Medicine, Gainesville, USA; 2 Internal Medicine, Al-Balqa' Applied University, As-Salt, JOR; 3 Pulmonary, Critical Care, and Sleep Medicine, University of Florida College of Medicine, Gainesville, USA

**Keywords:** clinical case report, vascular air embolism, air embolism, spontaneous air ambolism, covid – 19

## Abstract

A 72-year-old male with a complex medical history, including chronic obstructive pulmonary disease (COPD), hypertension, atrial fibrillation, and a recent COVID-19 infection, presented to the emergency department with shortness of breath and chest pain. Physical examination revealed stable vital signs but notable bilateral decreased air entry and diffuse wheezing. A computed tomography angiogram (CTA) of the chest confirmed a small to moderate volume of air embolism within the main pulmonary artery and right ventricle, with no evidence of pulmonary embolism. The air embolism was suspected to have been introduced during a contrast injection for the CT scan, as no other iatrogenic factors, recent invasive procedures, or history of lung trauma were present. Initial management included repositioning the patient to a supine position and administering 100% oxygen, which was critical in stabilizing his condition. Despite the ongoing symptoms of shortness of breath, the patient's condition improved with supportive care focused on managing COPD exacerbation. Spontaneous air embolism without decompression sickness or prior instrumentation is exceptionally rare, particularly in a post-COVID-19 patient, making this case notable. It highlights the critical need for prompt recognition, thorough evaluation, and appropriate management of air embolism in complex medical scenarios to prevent life-threatening complications. This case also underscores the importance of considering iatrogenic causes, such as contrast injection, in the differential diagnosis, especially following recent imaging studies.

## Introduction

Air embolism, also known as gas embolism, is a relatively rare medical condition, predominantly associated with iatrogenic causes [1]. It occurs when air or gas enters the circulatory system, leading to potentially severe consequences [2]. Understanding the point of entry and the underlying mechanisms involved allows for the categorization of air embolism into two main types: venous and arteria [2,3]. Venous pulmonary air embolism, in particular, is of significant concern due to its potential to cause life-threatening complications. Such complications can manifest as cardiogenic compromise, ultimately resulting in fatal outcomes [4]. Thus, it is imperative to recognize and promptly manage air embolism to mitigate its dire consequences. 

## Case presentation

We present the case of a 72-year-old man with a complex medical history, including chronic obstructive pulmonary disease (COPD), hypertension, atrial fibrillation, syndrome of inappropriate antidiuretic hormone secretion (SIADH), hypothyroidism, and a previous symptomatic COVID-19 infection three months prior to his emergency department admission. The patient's primary complaints upon presentation were shortness of breath and chest pain. The chest pain was described as central, non-radiating, accompanied by minimal productive cough, and notably, the patient denied experiencing palpitations or diaphoresis. Physical examination revealed stable vital signs, with oxygen pulse saturation at 94% while receiving three liters per minute of supplemental oxygen via nasal cannula. The patient remained alert and oriented to time, place, and person. Lung examination indicated bilateral decreased air entry and diffuse wheezing, with hyper-resonance on percussion. However, the examination revealed no crepitation or crackles. 

Initial laboratory investigations are demonstrated in Table 1. Viral molecular/respiratory polymerase chain reaction (PCR) testing returned negative results for COVID-19, influenza A/B, and respiratory syncytial virus (RSV). 

**Table 1:** Lab values. μL: microliter, ng/mL: nanograms per milliliter, mmol/L: millimoles per liter, mg/dL: milligrams per deciliter, pg/mL: picograms per milliliter, pg/mL: picograms per milliliter, BNP: B-type natriuretic peptide.

Laboratory	Value	Reference range
WBC	5.3	4-10 x 10^3^/L
Procalcitonin	0.01	-
Serum bicarbonate	40	22-30 mmol/l
Creatinine	1.16	0.51-1.18 mg/dl
BNP	85	<100 pg/ml
Troponin I HS	<0.6	<20 pg/ml

**Figure 1:**
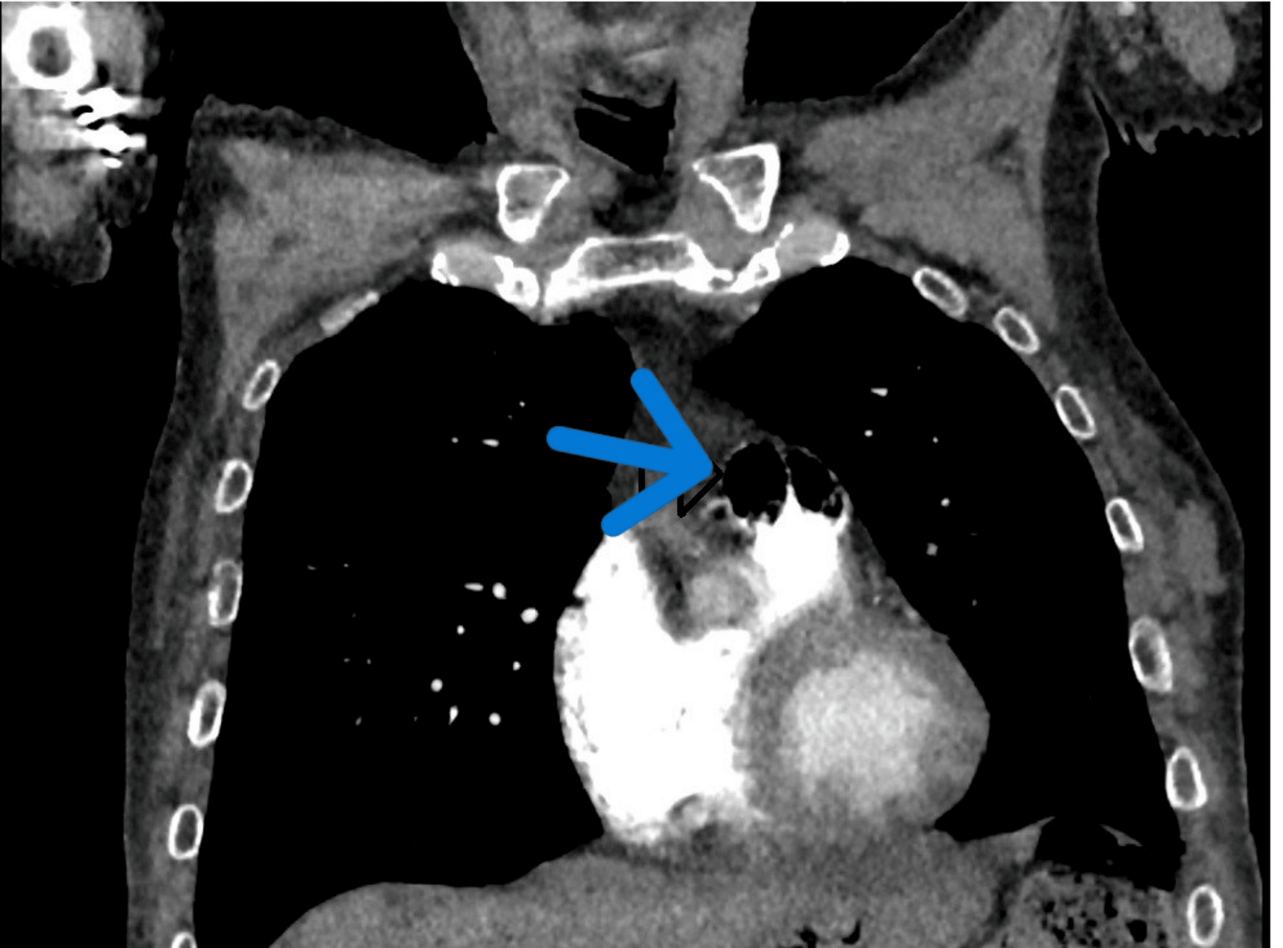
Sagittal view of computed tomography (CT) scan of the chest, showing a small to moderate volume of air embolism (indicated by the arrow) within the main pulmonary artery and right ventricle.

**Figure 2:**
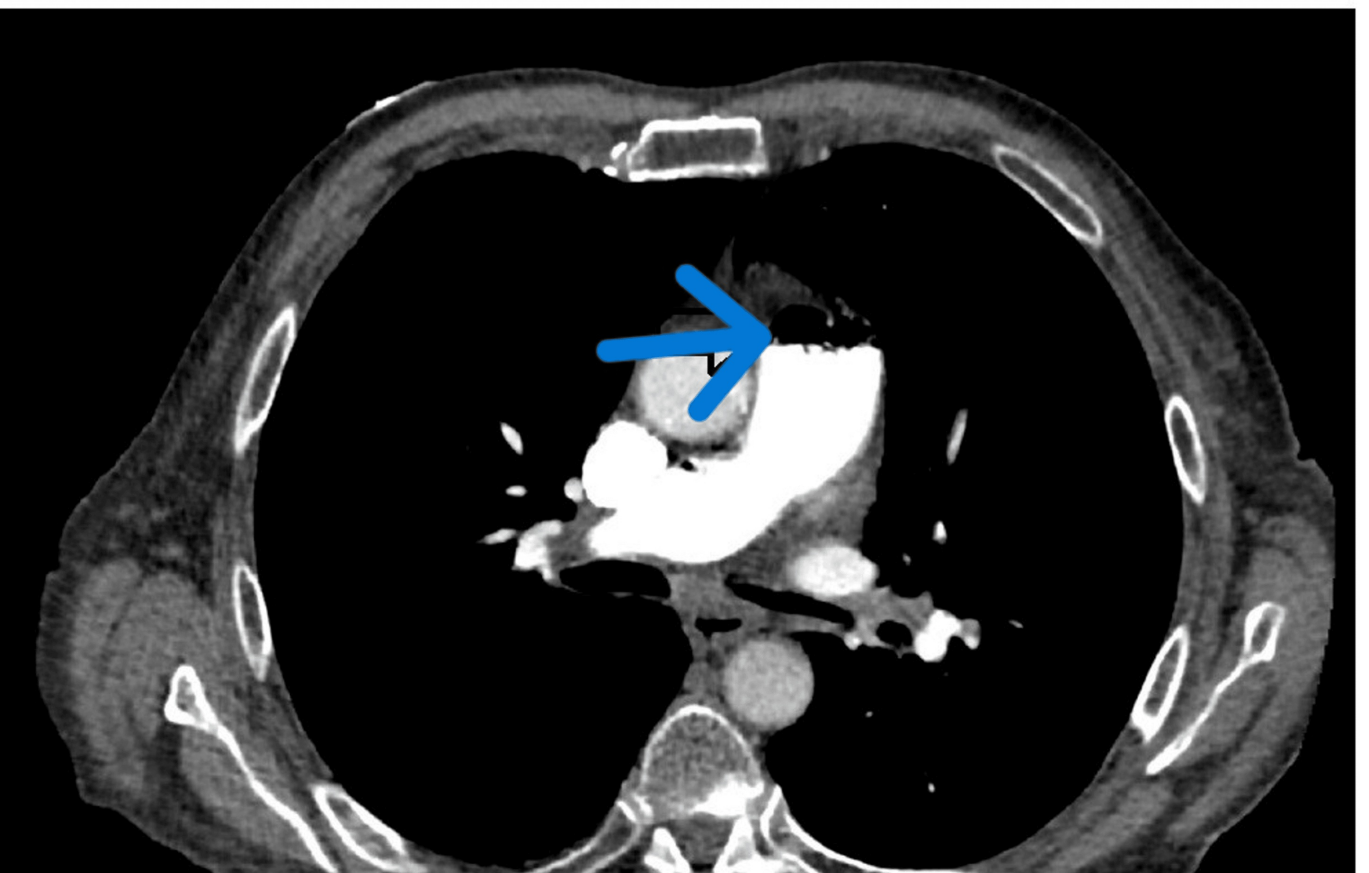
Axial view of a computed tomography (CT) scan of the chest, showing a small to moderate volume of air embolism (indicated by the arrow) within the main pulmonary artery and right ventricle.

Chest X-ray findings were unremarkable, with no evidence of infiltrations, pneumothorax, or pleural effusion. However, a computed tomography (CT) of the chest revealed the presence of a small to moderate volume of air embolism within the main pulmonary artery and right ventricle (Figures 1, 2). Furthermore, old posterior left upper lobe opacities were observed, which remained unchanged when compared to previous chest CT scans.

The differential diagnosis for the presented case includes a broad spectrum of possibilities given the patient's complex medical history and the potential overlap of symptoms. Firstly, considering the respiratory symptoms of shortness of breath and chest pain, pulmonary causes such as exacerbation of chronic obstructive pulmonary disease (COPD), pneumonia, pneumothorax, or pleural effusion need to be explored. Additionally, given the patient's history of recent COVID-19 infection, consideration should be given to pulmonary embolism or post-COVID-19 complications such as pulmonary fibrosis or thromboembolic events.

Cardiovascular etiologies, including acute coronary syndrome, heart failure exacerbation, or arrhythmias such as atrial fibrillation, should be considered, given the patient's history of hypertension and atrial fibrillation.

In the context of the patient's complex medical history and the incidental finding of air embolism, other potential causes, such as iatrogenic factors, need to be carefully evaluated. In this case, the possibility of air being introduced during the contrast injection for the CT scan should be considered. The patient had no recent procedures involving instrumentation, such as central venous catheter insertion or surgical interventions. Moreover, there was no history of lung trauma.

Given the rarity of spontaneous air embolism without decompression sickness or instrumentation, other less common etiologies considered include paradoxical air embolism through intracardiac shunts or traumatic injury to the vasculature.

Throughout the treatment course, the patient remained hemodynamically stable. High-flow oxygen therapy was initiated, and the patient was repositioned to a supine position. Despite ongoing symptoms of shortness of breath and chest discomfort, an interventional radiology consultation was conducted. However, it was concluded that suction thrombectomy for the observed air in the pulmonary artery and right ventricle would not benefit the patient. Consequently, the treatment approach shifted towards managing COPD exacerbation with prednisone 40 mg, azithromycin, and DuoNeb (ipratropium and albuterol) nebulizer.

Outcome and follow-up: The patient showed notable improvement in symptoms, reverting to his usual oxygen requirements. Upon discharge, he underwent evaluation by his primary care provider. Subsequent imaging studies were not pursued.

## Discussion

Air embolism is a rare condition often linked to activities or events such as trauma, surgical procedures, diving, and aviation. The pathophysiological consequences can include heightened pulmonary artery pressures, increased ventilation-perfusion mismatch, and right ventricular failure. The severity of the condition is influenced by factors such as the amount of gas, the rate at which it enters the system, the type of gas involved, and the patient's position at the time of the embolism [5].

The most frequent cause of air embolism is via a non-iatrogenic phenomenon that occurs during diving. A swift change in ambient pressure causes dissolved gases, primarily nitrogen, to come out of the solution and form bubbles in the arterial and venous systems. This is known as decompression injury [6].

Iatrogenic causes of air embolism are typically due to the introduction of ambient air into the vasculature via instrumentation. Air embolisms have been documented in literature to be associated with the implantation of cardioverter-defibrillator (ICD) [7], use of extracorporeal membrane oxygenation (ECMO) [8], inadvertent venous injection of 150 ml of air using contrast power injector during computed tomography (CT) [9], surgical correction of atrial septal defect [10], lung trauma, severe head injury after high-velocity motor vehicle accident [11], or pulmonary vein injury in the setting of percutaneous transthoracic needle biopsy (PTNB) [12]. To our knowledge, there is no clear association between remote COVID-19 infection and air embolism. However, a COVID-19 patient undergoing ECMO and contrast imaging procedures has reported a case of pulmonary air embolism [8]. 

In arterial circulation, air bubbles can cause blockages, which are facilitated by thromboinflammatory changes in the complement and hemostatic systems. This can lead to vasogenic edema, increased blood viscosity, activation of leukocytes and platelets, and a rise in intracranial pressure (ICP). These factors collectively reduce cerebral perfusion pressure and disrupt the blood-brain barrier (BBB). Air-mediated activation of C3 is associated with the release of proinflammatory cytokines. Additionally, air can activate the coagulation cascade through both complement-dependent and independent mechanisms [13].

As the bubble travels into progressively smaller blood vessels, it undergoes a phenomenon called 'slug flow,' transforming into a Taylor bubble-an elongated bubble that spans the vessel's diameter. This can interrupt blood flow by starting and stopping its movement. The recirculation at the bubble's tail can lead to the co-location of fibrinogen, platelets, red blood cells, and white blood cells. A clot forms as platelets become activated, along with clotting factors and fibrinogen accumulating at the tail. This can eventually cause the bubble to come to a stop in a small vessel, leading to obstruction and infarction [14]. Complement activation also contributes to additional platelet activation and clot formation. It's important to note that the extent of complement activation is not influenced by the type of gas involved; air, pure oxygen, and pure nitrogen all activate complement to the same extent [15].

Initial treatment involves positioning the patient appropriately. In cases of venous air embolism, the Durant maneuver is employed, which entails placing the patient on the left side with the head lower than the feet, known as the Trendelenburg position [16]. Durant's position helps by decreasing the obstruction of air emboli in the brain and alleviating the 'air lock' effect in the right heart [15]. For arterial air embolism, the Durant maneuver can be harmful as it may increase intracranial pressure (ICP) and exacerbate cerebral air embolism. Therefore, it is recommended for these patients to lie in a supine position [15]. Definitive treatment starts with inhalation of 100% oxygen, which creates a steep concentration gradient between the blood and alveoli, thereby speeding up the removal of nitrogen through the pulmonary system [16]. The subsequent step is hyperbaric oxygen therapy (HBOT), which functions by raising ambient pressure. This increase enhances the solubility of gases such as oxygen and nitrogen in the plasma, allowing them to be expelled when the patient breathes out [17]. Hyperoxia induced by HBOT also enables a higher amount of oxygen to dissolve in both plasma and tissues [18]. HBOT also minimizes leukocyte adherence to injured endothelium, which helps prevent cerebral edema by reducing microvascular permeability and strengthening the blood-brain barrier (BBB) [19]. HBOT has successfully treated air embolism as late as 52 hours after the development of neurologic symptoms [20].

Achieving euvolemia can help reduce significant pressure gradients between the pulmonary and systemic circulations, potentially lowering the risk of venous air embolism converting to arterial air embolism. Hemoconcentration, indicated by elevated hematocrit levels, has been linked to significantly higher rates of residual neurological symptoms [21]. Dextran-containing solutions should be avoided, as even small amounts of glucose can lead to increased lactate production and intracellular acidosis, which are associated with worse neurological outcomes [22].

Spontaneous air embolism without the presence of decompression sickness or instrumentation, such as was the case in our patient, is exceedingly rare and limited to individual case reports. One such case was the development of a spontaneous cerebral air embolism postulated to be secondary to a complication of a cholesteatoma triggered by the change in air pressure during a yoga session [23].

## Conclusions

This case underscores the complexity of diagnosing and managing spontaneous air embolism in patients with a multifaceted medical history. The patient presented with acute respiratory distress and chest pain, symptoms that could be attributed to a variety of underlying conditions. Despite initial suspicions, the association of air embolism with a previous COVID-19 infection was deemed unlikely due to the timing of the infection and the typical course of air embolism. The likely etiology, in this case, was the introduction of air during a contrast injection for the CT scan, highlighting a critical area for clinical vigilance.

Prompt recognition and management, including positioning maneuvers and oxygen therapy, were essential in mitigating potential complications. This case emphasizes the importance of considering iatrogenic causes in the differential diagnosis of air embolism, especially in patients with recent imaging studies. It also highlights the need for thorough evaluation and a high index of suspicion in complex clinical scenarios to ensure timely and effective treatment.
